# Radiation dosimetry of [^18^F]VAT in nonhuman primates

**DOI:** 10.1186/s13550-015-0149-4

**Published:** 2015-12-10

**Authors:** Morvarid Karimi, Zhude Tu, Xuyi Yue, Xiang Zhang, Hongjun Jin, Joel S. Perlmutter, Richard Laforest

**Affiliations:** Department of Neurology, Washington University Medical School, St. Louis, MO 63110 USA; Mallinckrodt Institute of Radiology, Washington University Medical School, St. Louis, MO USA; Department of Neurobiology, Washington University Medical School, St. Louis, MO USA; Department of Physical Therapy, Washington University Medical School, St. Louis, MO USA; Department of Occupational Therapy, Washington University Medical School, St. Louis, MO USA

**Keywords:** [^18^F]VAT, PET, Dosimetry, Primate, Vesicular acetylcholine transporter

## Abstract

**Background:**

The objective of this study is to determine the radiation dosimetry of a novel radiotracer for vesicular acetylcholine transporter (−)-(1-((2R,3R)-8-(2-[^18^F]fluoro-ethoxy)-3-hydroxy-1,2,3,4-tetrahydronaphthalen-2-yl)piperidin-4-yl)(4-fluorophenyl)-methanone ([^18^F]VAT) based on PET imaging in nonhuman primates. [^18^F]VAT has potential for investigation of neurological disorders including Alzheimer’s disease, Parkinson’s disease, and dystonia.

**Methods:**

Three macaque fascicularis (two males, one female) received 185.4–198.3 MBq [^18^F]VAT prior to whole-body imaging in a MicroPET-F220 scanner. Time activity curves (TACs) were created from regions of interest (ROIs) that encompassed the entire small organs or samples with the highest activity within large organs. Organ residence times were calculated based on the TACs. We then used OLINDA/EXM 1.1 to calculate human radiation dose estimates based on scaled organ residence times.

**Results:**

Measurements from directly sampled arterial blood yielded a residence time of 0.30 h in agreement with the residence time of 0.39 h calculated from a PET-generated time activity curve measured in the left ventricle. Organ dosimetry revealed the liver as the critical organ (51.1 and 65.4 μGy/MBq) and an effective dose of 16 and 19 μSv/MBq for male and female, respectively.

**Conclusions:**

The macaque biodistribution data showed high retention of [^18^F]VAT in the liver consistent with hepatobiliary clearance. These dosimetry data support that relatively safe doses of [^18^F]VAT can be administered to obtain imaging in humans.

## Background

Cholinergic neurotransmitters play an important role in brain function. Striatal cholinergic interneurons modulate the function of dopaminergic and glutamatergic inputs to striatum from other brain regions including the substantia nigra, thalamus, and cortex. Cholinergic projections also target other brain regions including the hippocampus, cerebellar vermis, and thalamus. Mounting evidence implicates abnormalities in cholinergic systems in numerous neurological conditions such as Parkinson’s disease (PD), dystonia, and Alzheimer’s disease (AD). Alterations in cholinergic function could contribute to involuntary movements that develop in people with PD called l-dopa-induced dyskinesias (LID). Indeed, selective nicotinic acetylcholine receptor partial agonists can reduce LID, while extending the duration of motor benefits in nonhuman animal models of PD [1, 2]. Cholinergic neurons in the pedunculopontine nucleus (PPN) and their thalamic efferent terminals could be involved in postural instability in PD [3]. Furthermore, changes in nicotinic acetylcholine receptors may correlate with cognitive dysfunction in PD [4, 5]. The well-known clinical response to anticholinergic medications in dystonia suggests pathologic involvement of cholinergic neurons. In fact, one genetic mouse model of dystonia has increased endogenous cholinesterase activity in striatum with impaired long-term depression that can be improved by blocking acetylcholine receptors [6]. Finally, the severity of cognitive dysfunction in AD correlates with loss of cholinergic neurons in CNS [7]. Indeed, the basal forebrain has showed marked loss of cholinergic neurons in AD autopsy studies [8].

In vivo measurement of cholinergic neurons could be critical for understanding the pathophysiology of these neurologic conditions. A PET radiotracer that permits quantification of loss of cholinergic neurons would provide a useful tool for assessing the pathophysiological correlates of cognitive and motor symptoms. Measurement of the vesicular cholinergic transporter (VAChT) has the potential to provide means to quantify cholinergic neurons. VAChT is expressed in presynaptic cholinergic terminals. We have preclinical data demonstrating the potential of (−)-(1-((2R,3R)-8-(2-[^18^F]fluoro-ethoxy)-3-hydroxy-1,2,3,4-tetrahydronaphthalen-2-yl)piperidin-4-yl)(4-fluorophenyl)-methanone ([^18^F]VAT) as a VAChT radiotracer that has high brain uptake with excellent selectivity and specificity [9, 10]. The objective of this study is to calculate human radiation dosimetry estimates of [^18^F]VAT based on nonhuman primate studies. This will permit us to better estimate allowable [^18^F]VAT activity for use in human PET imaging.

## Methods

### Radiopharmaceutical preparation

The radiosynthesis for [^18^F]VAT was accomplished using a two-step procedure in a Good Manufacturing Practice (GMP) facility. We prepared [^18^F]fluoroethyl tosylate using an Eckert and Ziegler Modular-Lab system in the first step [11–13]. We synthesized [^18^F]VAT using a GE TRACERlab FX-N module as [^18^F]fluoroethyl tosylate reacted with the corresponding enantiopure phenol VAT precursor. The final product was purified by a reverse phase HPLC prior to formation of the injection dose using 10 % of ethanol in saline. The radioactive dose was authenticated by a quality control analytical HPLC system.

All procedures were approved by Animal Studies Committee of Washington University in St. Louis.

### Anatomical MRI

Each animal was anesthetized as described above and underwent a head, chest, abdominal, and pelvic T1-weighted MR and T2 with a Siemens 3T Trio scanner using a knee coil at least a week apart from the PET session. We used the MRIs as a guide to identify the location of the critical anatomical structures including the brain, heart, lungs, liver, gallbladder, spleen, kidneys, large and small intestines, bladder, and gonads.

### Nonhuman primate radiation dosimetry

Two males and a female macaque fascicularis underwent whole-body scan in a Siemens MicroPET-F220. The female macaque was scanned twice. The macaques were prepared as previously described [14, 15]. The macaques were fasted overnight before each study. Each animal was initially anesthetized with ketamine 10–15 mg/kg i.m. They also received glycopyrrolate i.m. to reduce secretions. A 20-gauge plastic catheter was inserted into a limb vein to permit radiotracer; another 20-gauge plastic catheter was inserted into a femoral artery for arterial blood sampling (first scan of the female macaque); and a soft-cuffed endotracheal tube was inserted into the trachea to permit ventilation with isoflurane to maintain anesthesia. Lacrilube was placed into the animal’s eyes to protect the corneas, and eyelids were taped shut. Pulse, end-tidal PCO_2_, and rectal temperature were monitored. We used surgical tape to secure the head position of the anesthetized animal. The torso was wrapped in a warming blanket, and surgical tape was used to keep the torso in place. The transaxial positioning of the scanning bed is computer controlled within 0.2-mm tolerances permitting repeated repositioning of the animal for transaxial emission and transmission scans at multiple levels.

### Data acquisition

**Fig. 1:**
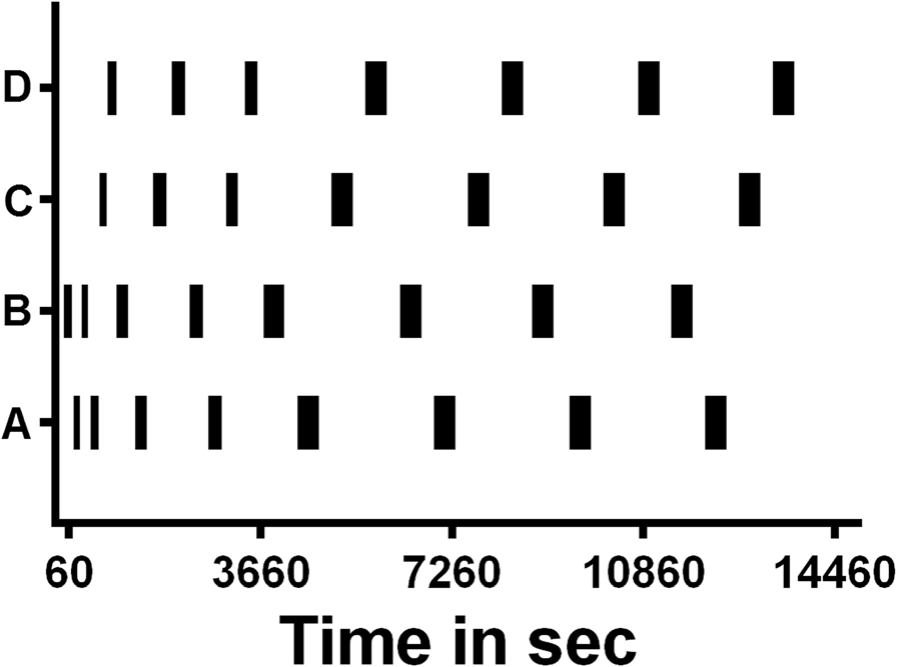
Seven to eight successive PET scans of increasing duration were obtained to cover *A* (whole brain), *B* (heart and lungs), *C* (liver, gallbladder, and kidneys), and *D* (urinary bladder, small and large intestines)

The animals weighed 7.3 (male), 6.4 (male), and 4.5 (female) Kg and received 185.4, 187.2, and 198.3 MBq of [^18^F]VAT, respectively, with >74 GBq/μmol specific activity. The female animal was scanned twice and received 197.6 MBq for the second scan. Biodistribution data of [^18^F]VAT were obtained from sequential whole-body PET images. Prior to radioligand injection, we collected four 30-min transmission scans using a Co-57 point source to cover four body sections: *A* (whole brain), *B* (heart and lungs), *C* (liver, gallbladder, and kidneys), and *D* (urinary bladder, small and large intestines). Seven to eight successive PET scans were done for each of the four sections (Fig. 1) immediately following [^18^F]VAT injection. Arterial blood samples were obtained for the first study in the female animal over a total scanning time of approximately 4 h. Most of the samples were taken in the first 3 min to ensure adequate description of the arterial time activity curve. Total radioactivity content in each blood sample was measured in a well counter cross-calibrated with the MicroPET-F220.

### Data analysis

We coregistered each emission scan to the corresponding transmission scan and could further confirm that there were no changes in the positioning between transmission and emission scans. Sinogram data were collected after [^18^F]VAT injection and reconstructed using filtered back projection with a ramp filter at Nyquist frequency along with correction for scatter, random, attenuation, and dead time. Reconstructed resolution was <2.0-mm full width half maximum for all three dimensions at the center of the field of view. PET image counts were calibrated to a dose calibrator to convert measured PET uptake to MBq of F-18. For some organs (whole brain, heart, lungs, urinary bladder), regions of interest (ROIs) were drawn to entirely encompass the target organ using ASIPro VM^™^ MicroPET analysis software (Siemens PreClinical Solutions, Knoxville, TN). For others (liver, spleen, small intestines, kidney, and gallbladder), we could not delineate a region large enough without including neighboring organs. Hence, we chose several small ROIs with the highest radioactivity within the target organ. The appropriateness of these ROIs was confirmed by comparison with the anatomical MRIs. The average radioactivity concentrations of these ROIs were multiplied by the entire organ weight to give an estimate of the radioactivity within the total organ. This approach will lead to slight underestimation of the values. However, as we are sampling the subregions with the most uptake and generalizing it to the entire organ, this underestimation is overcorrected.

The calculated activity in the whole organ was plotted as a function of time to yield the organ time activity curves. Organ residence times were computed from the analytical integration of the multi-exponential fit with two exponentials on the decay corrected time activity curves using GraphPad Prism6 software.

For the bladder, the cumulative activity was fitted by an uptake function of the form: *F*(*t*) = *A*0*(1-exp(−*A*1.t) where *A*0 and *A*1 were fitting parameters representing the filling fraction and filling constant. These parameters were entered in OLINDA MIRD bladder voiding model under the assumption that the bladder is emptied every 2 h. For the small intestines, we used the linear fit for the decay uncorrected time activity curve. The area under the curve was calculated with the trapezoid method.

The blood time activity curve for nonhuman primate data was integrated using the trapezoid method to yield the blood residence time. The blood activity was converted to percent injected dose (198.3 MBq) in the animal assuming a density of 1 g/cm^3^ and blood volume of 8 % of the animal body weight [16]. Finally, 5 % of the blood volume [17] was assumed to be present in the left ventricle at all times.

The maximum theoretical residence time (*T*_1/2_/ln(2) or 2.64 h for F-18) minus the sum of measured residence times was assigned to the remainder of the body as nonspecific activity. No loss of urine or fecal matter was observed in these animals during the scans. The calculated residence times were scaled following this equation: scaled *T*(human) = *T*(animal) × [(*M*_organ (human))/(*M*_total (human))]/[(*M*_organ (animal))/(*M*_total (animal))]. We used human male and female organ weights in OLINDA/EXM [18] for the adult male and adult female models. We applied percentage body weight for male and female macaque fascicularis for the kidney, liver, spleen, and heart as reported previously [19]. We used the gallbladder, bladder, and intestine percentage body weights of male and female macaque fascicularis we had euthanized in the past. We calculated macaque male and female organ weights for the brain and lungs based on their measured volume assuming a density of 1 g/cm^3^. The scaled human residence times were entered in the program OLINDA/EXM 1.1 for F-18 following the adult human male and female anthropomorphic models.

## Results

### Nonhuman primate biodistribution

**Fig. 2:**
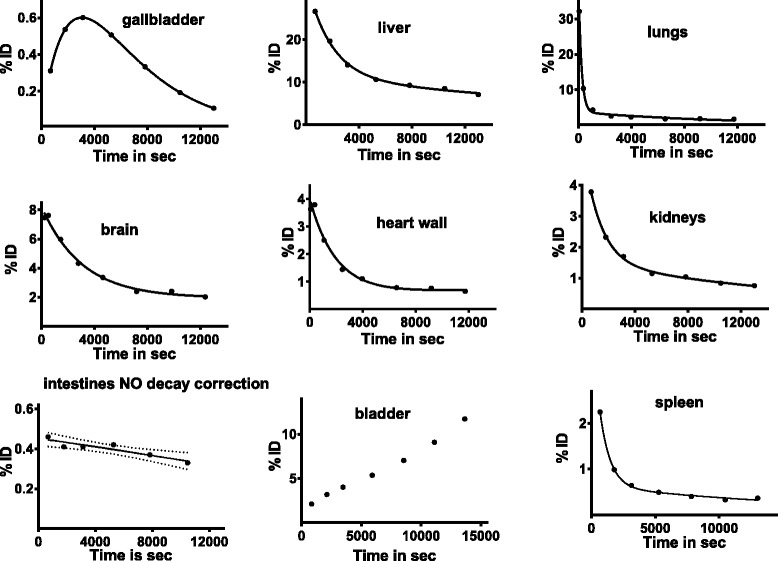
Tissue activity curves with activity expressed in percentage of injected dose per organ

Figure 2 shows the time activity curves compiled from sequential imaging of a male macaque. The time activity data were fitted with a function composed of single or two exponentials. Least square minimization was performed to find the best fitting parameters. We observed a predominant accumulation of the activity in the liver, brain, heart wall, and kidneys with initial fraction at ~30, ~8, 4, and 4 % of the injected dose, respectively, followed by a slow clearance.

### Residence times and dosimetry from nonhuman primate biodistribution data

**Fig. 3:**
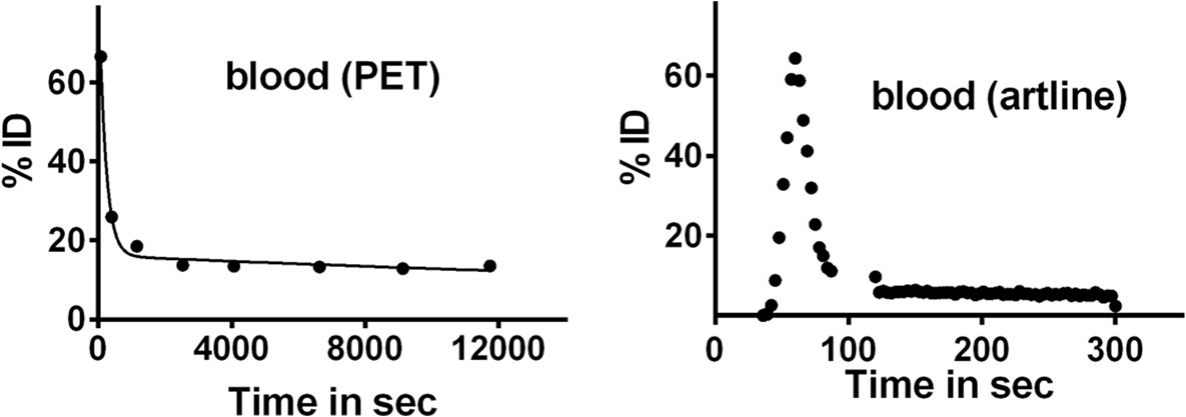
Tissue activity curves for blood based on PET-measured activity in the left ventricle and arterial blood sampling. Note that PET cannot capture the rapid rise of blood activity occurring in the first 50 s following the iv [^18^F]VAT injection

We averaged the values of the two measurements for the residence time in the female macaque (Table 1). The low standard deviation suggests good reproducibility. We had only one measurement of the male gallbladder as the top slices of the abdomen were not captured in the PET scan of the second male macaque. Directly collected arterial blood sampling was done only for the female monkey as shown in Fig. 3 and yielded a residence time of 0.30 h. This was in agreement with the residence time of 0.39 h calculated from a PET-generated time activity curve measured in the left ventricle.

**Table 1:** Organ residence times in hour

Organ	Residence time (h)	Residence time (h)
	Average two males ± SD	Average two measures in one female ± SD
Liver	0.32 ± 0.03	0.33 ± 0.12
Heart wall	0.05 ± 0.004	0.03 ± 0.004
Brain	0.09 ± 0.02	0.11 ± 0.01
Lungs	0.08 ± 0.04	0.05 ± 0.005
Gallbladder	0.01	0.01 ± 0.0008
Kidneys	0.04 ± 0.0002	0.04 ± 0.01
Spleen	0.02 ± 0.00004	0.02 ± 0.002
Small intestine	0.06 ± 0.02	0.04 ± 0.008
Entire blood	0.44 ± 0.06	0.39 (PET)
		0.30 (art line)
Urinary bladder	0.08 ± 0.04	0.08 ± 0.01
Blood assigned to the left ventricle	0.02	0.02
Remainder of the body	1.76	1.30

We calculated organ dosimetry based on its residence times with OLINDA/EXM 1.1 for the human adult male and female model as presented in Table 2. The effective dose and effective dose equivalent for human male and female adults are presented in Table 3.

**Table 2:** Extrapolated human radiation dose estimates for [^18^F]VAT in microgray/megabecquerel

Target organ	Males (μGy/MBq)	Females (μGy/MBq)
Adrenals	15	18.7
Brain	29.1	40.9
Breasts	8.6	10.7
Gallbladder	*31.1*	*53*
Lower large intestine	12.6	15.6
Small intestine	34.1	30.5
Stomach	12.6	15.7
Upper large intestine	15.9	18.4
Heart	40.2	34.6
Kidneys	28	35.8
Liver	*51.1*	*65.4*
Lungs	18.1	16.2
Muscle	10.1	12.5
Ovaries		16.4
Pancreas	15.3	19.1
Red marrow	10.4	12.7
Osteogenic cell	15	20
Skin	7.6	9.6
Spleen	26	32.8
*Testes*	9.1	
Thymus	10.8	13.2
Thyroid	9.7	11.2
Urinary bladder	33.7	52.3
Uterus		17.2
Total body	12.3	15.3

Data in italics represent critical organs

**Table 3:** Effective dose and effective dose equivalent for human from nonhuman primate biodistribution data

	Male	Female
Effective dose (μSv/MBq)	16	19
Effective dose equivalent (μSv/MBq)	20	25

## Discussion

The nonhuman primate measures may reasonably predict safe dosimetry levels for subsequent human exposures. These studies better predict human exposure dosimetry than rodent studies since metabolism may be quite different. For example, rodents do not have a gallbladder, and those radiopharmaceuticals that undergo liver metabolism may have gallbladder as a critical organ with a relatively high radiation exposure [20]. This would be completely missed in rodents whereas studies in nonhuman primates will capture this. Thus, these nonhuman primates provide a critical step that provides an additional margin of safety for radiopharmaceutical development and implementation in humans.

The availability of suitable radiopharmaceuticals for measuring VAChT in human brain is particularly important. VAChT may be involved in several diseases including dystonia, Parkinson’s disease, schizophrenia, and Huntington’s disease [21, 22]. These radiotracers also may provide measurements of target engagement for therapeutic interventions. [^18^F]VAT provides another radiopharmaceutical option that may have advantages over [^18^F]-FEOBV based upon better uptake in the brain [10].

## Conclusions

We used PET measures of radioactivity in organs and direct arterial blood sampling to calculate dosimetry exposure after the iv injection of [^18^F]VAT in nonhuman primates. The liver was the critical organ with radiation dosimetry of 51.1 μGy/MBq for males and 65.4 μGy/MBq for females. We calculated an effective dose of 16 and 19 μSv/MBq for male and female, respectively. Given the relatively low exposure to gonads and bone marrow (radiosensitive organs), we should be able to inject up to 764 MBq under the 21 CFR 361.1 guidelines (<50 mSv to any organ, <30 mSv to radiation sensitive organs).

### Statement of human rights

This article does not contain any studies with human participants performed by any of the authors.

### Statement on the welfare of animals

All applicable international, national, and institutional guidelines for the care and use of animals were followed. All procedures were approved by Animal Studies Committee of Washington University in St. Louis.
